# Integrative Mapping of Regulatory Variation in African American Hepatocytes Using Colocalization and MPRA: Toward Precision Drug Response

**DOI:** 10.21203/rs.3.rs-9611937/v1

**Published:** 2026-05-20

**Authors:** Carolina Clark, Guang Yang, Cristina Alarcon, Mrinal Mishra, Minoli A. Perera

**Affiliations:** Northwestern University; St. Jude Children's Research Hospital; Northwestern University; Northwestern University; Northwestern University

**Keywords:** Pharmacogenomics, African ancestry, hepatocytes, quantitative trait mapping, regulatory variation, precision medicine

## Abstract

**Background:**

Genomic datasets informing pharmacogenomic discovery often underrepresent individuals of African ancestry, limiting understanding of ancestry-specific regulatory variation and its implications for precision medicine. This gap hampers accurate gene regulatory modeling and equitable delivery of precision medicine.

**Results:**

We generated an expression quantitative trait loci (eQTL) dataset from 75 primary human hepatocytes derived from cadaveric livers of African American (AA) donors and integrated these data with public liver eQTL resources.

Cis-eQTL mapping identified 31,606 eQTLs (SNP–gene pairs), including regulatory variation in pharmacogenomic-relevant genes such as *CYP4F11, SLC47A1, SLC28A1*, and *GSTM3*. Of these, 17,585 were specific to the AA-hepatocyte dataset, including the clinically actionable gene *F5*, suggesting ancestry-specific regulatory mechanisms. These AA-specific eQTLs exhibited greater allele frequency differentiation than colocalized eQTL signals (mean Fst = 0.163 vs. 0.139, p < 2.2×10^−16^) and lower effect size correlation (ρ = 0.314 vs. 0.619). Colocalization analysis with GTEx and Broadaway liver datasets identified shared eQTL signals with GTEx, comprising 2,658 variants within 95% credible sets, indicating shared regulatory architecture. Integration with massively parallel reporter assay (MPRA) data further supported the functional relevance of prioritized variants, including loci within *GSTM3*, an important drug-metabolizing enzyme.

**Conclusions:**

By integrating ancestry-specific eQTL mapping, colocalization, and functional assays, this study improves the resolution of causal regulatory variants in hepatocytes. Expanding diverse population cohorts and incorporating functional assays in relevant tissue will be crucial to further disentangle the complex genetic architecture of hepatic gene regulation. Our findings expand the understanding of regulatory mechanisms underlying pharmacogenomic traits and advance precision medicine in admixed populations.

## BACKGROUND

Understanding the genetic regulation of gene expression is essential for interpreting the functional consequences of noncoding genetic variation. Expression quantitative trait loci (eQTL) mapping serves as a powerful tool to link genetic variants to gene expression changes, offering insights into disease biology, complex trait architecture, and potential therapeutic targets^1–4^. However, most publicly available eQTL resources, including the Genotype-Tissue Expression (GTEx) project, predominantly represent individuals of European ancestry and bulk tissue types^5^. This underrepresentation of diverse ancestries and relevant primary cell types poses a critical barrier to capturing the full landscape of regulatory variation and hinders the equitable translation of genomic research into clinical applications^6–8^.

The liver plays a central role in drug metabolism, lipid regulation, and overall metabolic homeostasis, making it a key tissue for studies of pharmacogenomics and complex diseases. Hepatocytes, the liver’s main parenchymal cells, play a central role in regulating drug clearance, bioavailability, and toxicity. Genetic variation in hepatocyte-specific regulatory elements can influence expression of key drug-metabolizing enzymes and transporters, ultimately affecting drug response^9,10^. Yet, relevant eQTL studies are largely limited to bulk liver samples from predominantly European ancestry donors^5,7,11^. This approach masks cell-type-specific signals and fails to capture ancestry-specific regulatory variants, both of which are essential for understanding physiological processes and disease in diverse populations^12–14^. Primary human hepatocytes offer a unique opportunity to study regulatory variation in a relevant cellular context with reduced confounding signals from cellular heterogeneity.

Prioritizing regulatory variants from eQTL datasets remains challenging due to the complexity of linkage disequilibrium (LD) patterns and the inherent noise in association-based analyses. Matched datasets, such as other quantitative trait mapping analyses or functional genomic assays, can aid in validating or prioritizing regulatory variants. Since many -omic data types (i.e., gene expression, DNA methylation) are dynamic and represent context-specific processes that vary across tissues, cell types, and genetic ancestries, it is important for matched datasets to be from same tissues and populations^15–17^. Because diverse hepatocyte eQTL datasets are extremely rare, there are limited opportunities to validate signals in perfectly matched systems. Nonetheless, publicly available datasets like GTEx liver provide a useful, though imperfect, benchmark for identifying shared signals across populations. In addition, massively parallel reporter assays (MPRAs) in pharmacologically relevant liver-derived cell types, such as HepG2, offer a powerful orthogonal strategy to prioritize functional regulatory variants by directly testing their effects on gene expression^18^.

In this study, we present an eQTL dataset derived from primary human hepatocytes obtained from African American (AA) cadaveric donors. This resource addresses a critical gap in eQTL research by increasing both cellular and ancestral diversity. Through a series of integrative analyses, we demonstrate the functional relevance of our hepatocyte eQTLs: they are significantly enriched in active regulatory regions, include a substantial number of eGenes and regulatory variants not identified in GTEx liver tissue, and show evidence of colocalization with GTEx liver eQTLs, highlighting both shared and context-specific signals. Furthermore, by integrating our eQTL results with MPRA data, we prioritize potentially causal regulatory variants. Together, these findings underscore the importance of ancestry- and cell-type-specific regulatory datasets for improving our understanding of gene regulation and its implications for disease biology in underrepresented populations.

## RESULTS

### AA-hepatocyte eQTLs are significantly enriched in active chromatin-state markers

**Figure 1:**
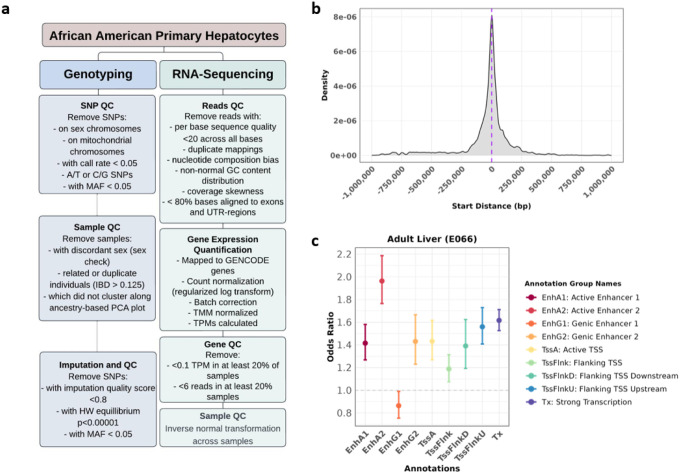
cis-eQTL analysis within African American primary human hepatocytes. (a) Schematic representation of the genotype and RNA-sequencing quality control pipelines. Following these QC steps, 73 individuals were included in subsequent eQTL analysis. (b) Density plot of the distance between eSNPs and the transcriptional start sites (TSS) of their associated eGenes. Cis-eQTLs were identified using tensorQTL with a 1MB window, adjusting for age, sex, genomic principal component 1, and 10 PEER factors. eGenes were defined using adaptive permutation and a q-value threshold of 0.10. Distances were calculated using MANE-select TSS annotations. (c) Enrichment analysis of African American hepatocyte cis-eQTLs within active regulatory regions defined by the ChromHMM 18-state model. Chromatin states classified as active (TssA, TssFlnkD/U, Tx, EnhG1/2, EnhA1/2) from Roadmap liver (E066) dataset were used as annotations. Enrichment was calculated relative to 1,000 matched null SNP sets, stratified by MAF, LD score, and TSS distance. Fisher’s exact test was used to assess significance, and p-values were corrected using the Bonferroni method.

Our eQTL analysis identified 31,006 unique eQTL variants (eSNPs), 31,606 significant SNP-gene pairs (eQTLs), and 679 eGenes within our AA-hepatocyte cohort (n = 73) (Supplemental Table 1). The distribution of significant eQTL distances from the TSS of their eGene shows the majority of eQTLs are concentrated near the TSS (Fig. 1B). This trend is consistent with the TSS being a critical regulatory region where transcription factors and other regulatory elements modulate gene expression. To further characterize the genomic locations of our significant eQTLs with respect to regulatory activity, we performed an enrichment analysis using active regulatory regions defined by Roadmap’s ChromHMM 18-state chromatin model within adult liver tissue (E066) and HepG2 cells (E118). The ChromHMM 18-state chromatin model uses multivariate Hidden Markov Modeling to consolidate epigenetic ChiP-seq data for six chromatin marks (H3K4me3, H3K4me1, H3K36me3, H3K27me3, H3K9me3, and H3K27ac) into biologically meaningful states^19^. AA-hepatocyte eQTLs were found to be significantly enriched in almost all active chromatin states within adult liver tissue and HepG2 cells (Fig. 1C, Figure S2A, Supplemental Table 2). Notably, genic enhancer 1(EnhG1) state was not significantly enriched in adult liver tissue (OR = 0.86, FDR = 3.51e-02) and was the only depleted active regulatory state across both tissues. The observed enrichment for AA-hepatocyte eQTLs was comparable in magnitude to that seen in GTEx liver eQTLs (Figure S2B-C, Supplemental Table 2).

### AA-hepatocyte eQTLs contain unique eGenes and eQTLs as compared to other liver eQTL datasets

**Figure 2:**
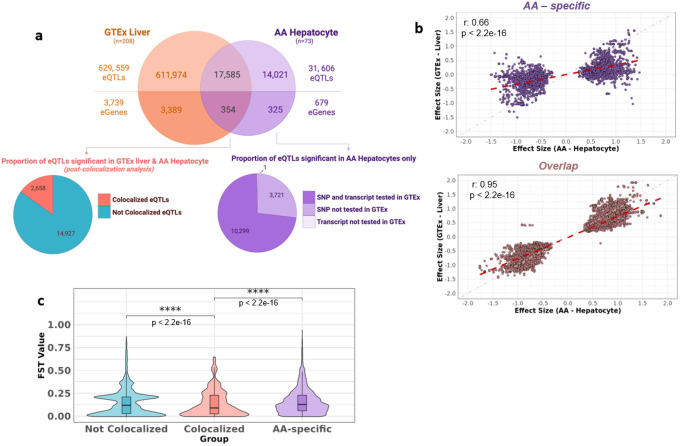
General comparison of African American hepatocyte eQTLs with GTEx liver eQTLs. (a) Venn diagrams summarize comparisons between significant results from African American (AA) hepatocytes and the GTEx liver dataset. The main diagram shows the overlap in eQTLs and eGenes between the two datasets. The bottom left diagram highlights how many of the overlapping eQTLs colocalized using the coloc method (posterior probability for colocalization, PP4 > 0.80, and credible set cumulative SNP.PP.H4 > 0.95). The bottom right diagram depicts the proportions of AA-specific eQTLs that were included in the GTEx-liver dataset and not significant vs. not tested in GTEx. (b) Scatterplots of absolute eQTL effect sizes (β) comparing GTEx liver and AA hepatocyte datasets. The left panel shows eQTLs unique to AA hepatocytes, while the right panel shows variants identified as significant in both datasets (overlapping eQTLs). Pearson’s rank correlation coefficient (r) and corresponding p-values are reported for each comparison. (c) Violin plots compare population differentiation (fixation index, FST) between eQTL groups: not colocalized, colocalized, and AA-specific. FST was calculated using allele frequencies from YRI and CEU populations in the 1000 Genomes Project. Statistical significance was assessed using the Wilcoxon rank-sum test.

We compared our AA-hepatocyte eQTLs to GTEx liver eQTL dataset (v8), which includes 629, 558 *cis*-eQTLs and 3,739 eGenes, to characterize potential population-specific and shared regulatory effects (Fig. 2A). Of the 679 eGenes identified in the AA-hepatocyte eQTLs, 354 were also eGenes in GTEx-liver (overlapping eGenes), 1 was not tested for association within GTEx, and 324 were tested for association within GTEx but were not identified as eGenes (AA-specific eGenes) (Fig. 2A). The overlapping eGenes constitute 17,585 overlapping eQTLs (55.6% of AA-hepatocyte eQTLs), while 14,020 eQTLs were unique AA-specific eQTLs (44.4% of AA-hepatocyte eQTLs). Similarly, we compared our AA-hepatocyte eQTLs to the Broadaway *et al*. liver eQTL dataset, which included 1,113,709 *cis*-eQTLs and 6,482 eGenes (Figure S3). Despite having a higher number of significant results, only 377 eGenes overlapped with AA-hepatocyte eQTLs, a marginal increase from the number of overlapping eGenes with GTEx. There were only 5,665 overlapping eQTL signals with Broadaway *et al*. (17.9% of AA-hepatocyte eQTLs), which is a notable decrease from the overlap with GTEx liver.

The magnitude of effect sizes between AA-hepatocyte and GTEx-liver was compared for AA-specific and overlapping eQTLs groups (Fig. 2B). While both groups showed a strong correlation (Pearson correlation, p-value 2.2e-16), indicating some degree of shared genetic regulation amongst both datasets, the correlation for AA-specific eQTLs (Pearson correlation = 0.66) was much lower than that of overlapped eQTLs (Pearson correlation = 0.95). Of note, the corresponding GTEx effect size for AA-specific eQTLs was significantly lower (Wilcoxon rank-sum test, p-value < 2.2e-16).

216 eGenes showed evidence of colocalization(PP4 > 0.8) between GTEx liver and AA-hepatocyte eQTL signals, comprising of 2,658 variants within their 95% credible sets and represent 15% of overlapping variants(Fig. 2A, Supplemental Table 3. Similarly, 180 eGenes showed evidence of colocalization between Broadaway et al. and AA-hepatocyte eQTL signals, comprising of 1,961 variants within their 95% credible sets and represent 34.6% of overlapping variants (Figure S3, Supplemental Table 4).

Fst scores were compared between variants found in GTEx colocalized and not-colocalized groupings, as well as AA-specific variants (Fig. 2C). Both comparisons were found to be statistically significant (Wilcoxon rank-sum test, p < 2.2e-16), indicating differences in population allele frequency differentiation between these eQTL groups. Notably, the colocalized group had the lowest average fst values among the three groupings.

**Figure 3:**
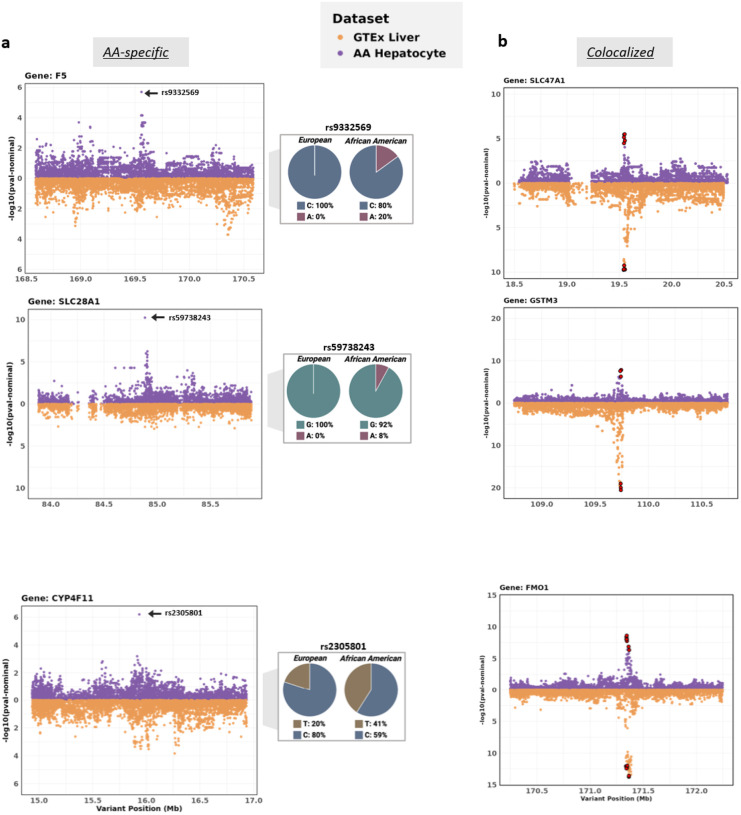
Representative eGenes from AA hepatocyte and GTEx liver eQTL datasets. To illustrate differences and similarities between the AA hepatocyte and GTEx liver eQTL datasets, we visualized the −log10(p-value) for SNP associations across representative eGenes in both datasets. For each gene, SNP associations are plotted by genomic position, with AA hepatocyte data in purple and GTEx liver data in orange. Representative genes were selected based on their known relevance to liver biology and grouped by their classification: (a) AA-specific eGenes – significant only in the AA hepatocyte dataset. The 1000 Genomes Phase 3 allele frequencies of the most significant eQTL variant in the AA dataset are displayed next to their respective eGene for both European (EUR) and African American (ASW) populations. (b) Colocalized eGenes – significant in both datasets and colocalization PP4 > 0.80. Red dots signify colocalized variants

We conducted a direct comparison between the results of GTEx liver data and our AA-hepatocyte dataset by plotting p-values for each eGene by dataset. Representative eGenes are shown in Fig. 3, grouped by whether they are AA-specific (Fig. 3A) or colocalized (Fig. 3B), both of which exhibited distinct patterns. The top AA-specific eQTLs are variants with differential allele frequencies between African and European populations, with allele frequencies derived from reference databases (e.g., the 1000 Genomes Project), emphasizing the importance of ancestry-specific regulatory variation. For example, Fig. 3A highlights *F5*, *SLC28A1*, and *CYP4F11* as AA-specific eGenes. The lead eQTL for *F5* (rs9332569:C > A, p-value = 1.99e-06) has a minor allele frequency (MAF) of 20% in African ancestry populations but is absent in Europeans. Similarly, the top hit for *SLC28A1* (rs59738243:G > A, p-value = 5.68e-11), a splice region variant, has an MAF of 8% in African populations and 0% in Europeans. In contrast, the lead eQTL for *CYP4F11* (rs2305801:T > C, p-value = 6.29e-07), a synonymous variant, shows differential minor allele frequency between populations: the T allele is less common, with a MAF of 20% in European populations and 41% in populations of African ancestry populations.

### Integrating HepG2 MPRA data helped prioritize causal regulatory variants

We used results from a HepG2 MPRA to further refine our AA-hepatocyte eQTL signals by identifying additional functional evidence supporting the regulatory role of specific variants. Of the 31, 006 eSNPs identified in our eQTL analysis, 2,335 (7.39%) were tested in the HepG2 MPRA. Of those, 168 (7.2%) were considered as active regulatory sequences in the MPRA. We integrated significant MPRA results (i.e., active regulatory sequences) with our eQTL findings and colocalization analysis with GTEx liver.

**Figure 4:**
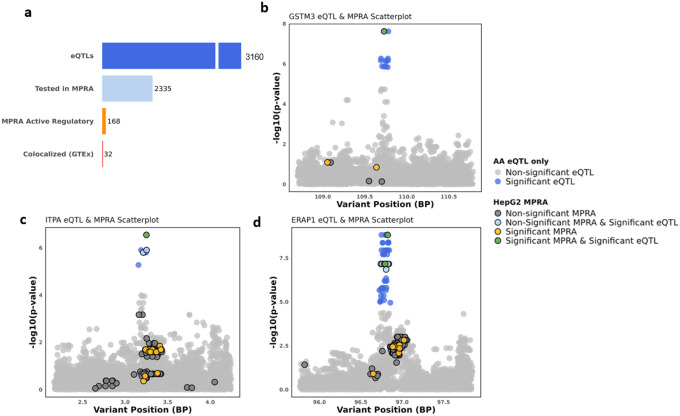
Integration of HepG2 MPRA results with AA hepatocyte eQTL analysis to functionally prioritize regulatory variants. Variants from a massively parallel reporter assay (MPRA) performed in HepG2 cells were integrated with our AA hepatocyte eQTL results. (A) A bar plot quantifies the sequential filtering of AA hepatocyte eQTLs through the MPRA dataset, showing the total number of eQTLs identified, the subset tested in the MPRA, those meeting MPRA significance thresholds, and the subset that also colocalized with GTEx liver eQTLs. (B-D) Dots without a border represent variants tested only in the AA eQTL analysis, while outlined dots represent variants tested in both the MPRA and our AA hepatocyte eQTL dataset. The color of each dot indicates whether the variant was significant in the MPRA only, eQTL only, or both. The following representative eGenes were selected for their association to liver biology and overlap with tested MPRA variants: (B) *GSTM3*(C) *ITPA* (D) *ERAP1*.

For eGenes with colocalized eQTL signals between our AA-hepatocyte dataset and GTEx liver, 32 significant MPRA variants overlapped with variants within the 95% credible sets of these colocalized signals. This overlap suggests that not only do these variants regulate gene expression in both datasets, but they also demonstrate direct regulatory activity in an independent MPRA assay. One example is *GSTM3*, a known phase II drug-metabolizing enzyme. As shown in Fig. 4A, a colocalized variant between the AA-hepatocyte dataset and GTEx liver also exhibited significant MPRA activity, with the direction of effect concordant with that observed in the AA-hepatocyte eQTL, providing additional evidence of its causal regulatory function. The convergence of colocalization and MPRA evidence strengthens the case for this variant as a functional driver of *GSTM3* expression.

For eGenes that did not have a colocalized eQTL signal between datasets but were eGenes in both the AA-hepatocyte and GTEx liver datasets, MPRA data helped resolve potential regulatory differences. In cases where no colocalized variant was identified, this indicated that different causal signals underlie gene regulation in the two datasets. However, when a significant MPRA variant overlapped with our top AA-hepatocyte eQTL, this provided additional evidence supporting the regulatory function of the AA-hepatocyte variant despite the lack of colocalization. A notable example is *ITPA*, an enzyme involved in purine metabolism with pharmacogenomic clinical relevance. As shown in Fig. 4B, the lead eQTL variant in our dataset (rs11699145) was also an eQTL in GTEx liver, but colocalization analysis suggested different causal variants across datasets. However, rs11699145 was identified as an active regulatory element in the MPRA with a consistent direction of effect relative to AA-hepatocyte eQTL, lending further support to its role as a functional regulatory variant in hepatocytes.

## DISCUSSION

Our findings demonstrate that AA-hepatocyte eQTLs are significantly enriched in ChromHMM active chromatin states, reinforcing their likely regulatory roles in gene expression within hepatocytes. The strong enrichment observed in the most active states within both adult liver tissue and HepG2 cells supports the functional relevance of these variants in transcriptional regulation. The concentration of eQTLs near the transcription start site (TSS) aligns with the well-established role of this region in gene regulation, where transcription factors and chromatin modifications play a crucial role in modulating gene expression.

Notably, the depletion of eQTLs within the EnhG1 chromatin state in adult liver tissue suggests a divergence in the regulatory landscape between primary hepatocytes and HepG2 cells. The EnhG1 state, characterized by associations with active histone modifications H3K4me1, H3K36me3, and H3K27ac, typically marks genic enhancers that drive transcription within transcribed regions^19^. While EnhG1 regions are active, their active regulatory markers are weaker compared to more canonical enhancers and active transcriptional start site regions^20^. In contrast, we observed strong enrichment of eQTLs in other active chromatin states such as EnhA (active enhancers), TssA (active transcription start sites), and Tx (strong transcription), which are generally associated with more potent regulatory activity. This pattern supports the notion that the AA-hepatocyte eQTLs are concentrated in the most functionally impactful regulatory regions, further reinforcing their biological relevance.

The selective depletion of EnhG1 in adult liver tissue, but not in HepG2 cells, suggests that AA-hepatocyte eQTLs are less likely to act on highly active genic enhancers in vivo and may instead influence gene expression through poised or fine-tuning regulatory mechanisms (e.g., EnhG2). This difference highlights the importance of cellular context when interpreting chromatin-based regulatory annotations and suggests that HepG2, while informative, may not fully recapitulate all regulatory landscapes present in primary hepatocytes. Further investigations into enhancer activity in hepatocytes from diverse populations could provide deeper insight into these differences and their potential implications for liver-specific gene regulation.

Our comparison between AA-hepatocyte eQTLs and GTEx liver eQTLs highlights the importance of population diversity and cellular context in genomic studies. GTEx liver eQTLs are derived from whole liver tissue while our eQTL dataset is specifically from primary human hepatocytes. The use of this single cell type may capture regulatory effects that are diluted in bulk tissue due to cellular heterogeneity. While GTEx v8 contains 208 liver samples and is more highly powered to identify significant eQTLs than the AA-hepatocyte dataset, only 12.4% of GTEx subjects are considered AA based on their genetic ancestry, and an even smaller subset (n = 18) contain both genotype and gene expression data in liver tissue^11^. As a result, identification of African-specific regulatory variation is limited within GTEx liver samples, and such signals may be overlooked in analysis.

The presence of AA-specific eGenes and eQTLs within our dataset, many of which involve variants with distinct allele frequencies between African and European populations, underscores the necessity of studying diverse populations to fully characterize genetic regulation. AA-specific eGenes exhibited unique peaks in the AA-hepatocyte data that were absent in GTEx liver and were often driven by variants with higher allele frequencies in African ancestry populations (Fig. 3A). For example, among the representative AA-specific eGenes highlighted in Fig. 3A, two loci (within *F5* and *SLC28A1*) harbored minor alleles that were entirely absent from European populations, precluding their detection in GTEx due to lack of statistical power. The *F5* variant (rs9332569) disrupts an ERα binding motif, a transcription factor implicated in hepatic homeostasis, while the *SLC28A1* variant (rs59738243) overlaps an active transcriptional start site in adult liver cells and enhancer-associated chromatin states as well as POL2 binding in HepG2 cells, supporting regulatory activity^19,21–24^. The third locus, within *CYP4F11*, demonstrated a more modest allele frequency difference (MAF = 41% in African ancestry vs. 20% in Europeans); in this case, the lack of replication in GTEx may instead reflect differences in linkage disequilibrium rather than the absence of the allele itself. Consistent with a putative regulatory role, the lead *CYP4F11* variant (rs2305801) is annotated as an active transcriptional start site in both adult liver and HepG2 cells^19^. In such cases, population-specific LD patterns may result in different associations across ancestries, as a tagging SNP present in both populations may only be in strong linkage with the causal variant in one. These examples highlight how both allele frequency differences and LD structure can contribute to the discovery of ancestry-specific regulatory variants. The AA-specific eQTLs showed lower effect sizes in GTEx data (Fig. 2B), reinforcing the idea that genetic effects observed in one population may not always replicate in another due to differences in genetic architecture, environmental exposures, or gene regulation.

In addition to these ancestry-specific signals, variants within credible sets for colocalized signals also demonstrate strong enrichment for liver-relevant regulatory annotations. At the *SLC47A1* locus, variants localize to active transcription start sites, enhancers, and DNase hypersensitive regions, and disrupt motifs for transcription factors such as IRF3, HNF3A, and SETDB1, all of which have established roles in hepatic gene regulation^20,25–28^. The *GSTM3* locus includes a 5′ UTR variant and active transcription start site annotations within liver-relevant cells, with motif disruptions affecting RXRA, SMAD3, AHR, and FXR, key regulators of liver metabolism, regeneration, and xenobiotic response pathways^21,29–33^. Similarly, variants at the *FMO1* locus map to enhancer regions in liver cells and disrupt motifs for PDX1, FOXA family members, and NR2F2, which are involved in liver development and metabolic control^21,34–37^. These observations provide complementary functional support for the regulatory potential of colocalized eQTL signals, suggesting that both ancestry-specific and shared associations converge on biologically meaningful regulatory mechanisms in hepatocytes.

Further stratification of eGenes based on GTEx colocalization status revealed key distinctions between regulatory patterns across datasets. Colocalized eGenes showed clear signal overlap, suggesting shared causal variants between populations. Variants in the colocalized group had the lowest average FST values, indicating smaller allele frequency differences between African (YRI) and European (CEU) populations. This is consistent with the GTEx liver dataset being composed predominantly of individuals of European ancestry, making colocalized variants more likely to reflect shared regulatory signals across ancestries. In contrast, the higher FST values observed among AA-specific and non-colocalized variants suggest greater population divergence and may reflect ancestry-specific regulatory effects that are underrepresented in GTEx. Collectively, these findings emphasize the value of integrating ancestry-specific datasets to improve the resolution of genetic regulatory architecture, as relying solely on European-biased reference datasets may obscure signals that are highly relevant for diverse populations.

We also compared our AA-hepatocyte eQTLs to a large liver eQTL meta-analysis by Broadaway *et al*., which included 1,183 liver samples of European ancestry. Unlike our study, which used RNA sequencing, Broadaway *et al*. employed expression microarrays, a technology with a narrower dynamic range and different sensitivity for detecting gene expression. Additionally, the statistical approaches differ: Broadaway *et al*. applied a genome-wide p-value threshold (p < 1×10^−^^5^) for their marginal eQTL analysis, whereas we used a hierarchical Benjamini–Hochberg correction at both the gene and variant levels. Despite Broadaway *et al*.’s larger sample size and greater number of identified eQTLs (1.11 million across 19,011 eGenes), only 5,665 of our 31,606 AA-hepatocyte eQTLs and 377 of 679 eGenes overlapped. Notably, of the 14,021 AA-specific eQTLs not found in GTEx, 12,469 were also absent from Broadaway *et al*., suggesting that ancestry (rather than statistical power) accounts for much of the unique signal in our dataset.

Our integration of MPRA data with eQTL and colocalization analyses provided valuable insights into the regulatory landscape of AA-hepatocyte eQTLs. In some cases, MPRA data reinforced colocalized eQTLs, offering independent functional validation of regulatory variants, as seen with *GSTM3*. However, for genes like ITPA, MPRA results helped resolve regulatory differences between datasets by highlighting distinct causal variants. Interestingly, in some cases, MPRA and eQTL results provided conflicting evidence, indicating a more complex regulatory landscape. For *ERAP1*, an inflammation-induced hepatokine linked to liver function and disease, we observed two conflicting patterns: (1) significant MPRA hits that were not identified as eQTLs, and (2) strong eQTL signals that did not show MPRA activity (Fig. 4C)^38–40^. This suggests that while MPRA is useful in identifying regulatory variants, it may not capture all eQTL mechanisms, possibly due to differences in assay conditions, context-dependent regulatory activity, LD structure, or limitations in MPRA sensitivity for certain enhancer elements. Notably, the MPRA data used here was generated in HepG2 cells rather than primary hepatocytes, which may further contribute to these discrepancies.

Several of the eGenes identified in our AA-hepatocyte eQTL dataset and highlighted in our results play well-established roles in hepatic drug metabolism and pharmacogenomic pathways, underscoring the functional relevance of this resource. Genes such as *CYP4F11*, *FMO1*, and *GSTM3* encode phase I and phase II drug metabolizing enzymes, which contribute to the oxidation, reduction, and conjugation of a wide range of xenobiotics. *CYP4F11* contains variants linked to altered warfarin dose requirements^41^. *FMO1* and *GSTM3* variants are linked to higher-dose adjusted olanzapine plasma concentrations and lower olanzapine exposure, respectively, implicating both genes in psychotropic drug metabolism/PK^42,43^. *GSTM3* variants are also associated with increased risk of side effects with cisplatin and cyclophosphamide chemotherapy treatments, as well as cancer progression^44–48^. *SLC28A1* and *SLC47A1* are membrane transporters that mediate hepatic uptake and efflux of nucleoside analogs and organic cations, respectively, with implications for drug bioavailability and toxicity^49–54^. *ITPA* and *ERAP1* have known pharmacogenomic associations, influencing response to thiopurine therapy and antigen processing, respectively, both of which may have downstream effects on immune response and treatment outcomes^38,55–57^. Notably, the coagulation factor *F5*, is primarily synthesized in the liver and is clinically relevant due to its role in thrombosis, interaction with anticoagulant therapies, and actionable Pgx drug label annotations^58,59^. Together, these findings emphasize the clinical and pharmacogenomic relevance of the variants and eGenes identified in our dataset and support their potential to inform precision medicine in underrepresented populations.

Despite the insights gained, our study has several limitations. First, the sample size of our AA cohort is relatively small, which may limit statistical power to detect eQTLs with small to moderate effect sizes and with low minor allele frequencies. Future studies with larger AA-hepatocyte datasets are needed to improve the resolution of ancestry-specific regulatory variants. Second, while GTEx liver tissue provides a useful reference for comparison, it consists of heterogeneous cell types, which may dilute hepatocyte-specific eQTL signals. The observed differences between our AA-hepatocyte eQTLs and GTEx liver eQTLs could be partly driven by this cellular heterogeneity rather than true ancestry-related differences in regulation. Of note, 80% of the liver is composed of hepatocytes. Third, our use of HepG2 MPRA data introduces challenges related to differences in cellular context, as HepG2 is a transformed cell line that may not fully recapitulate the regulatory environment of primary hepatocytes. Additionally, the MPRA library was constructed based on eQTLs identified in lymphoblastoid cell lines, which may have led to an underrepresentation of variants relevant to hepatocyte-specific gene regulation and African ancestry populations. Future MPRA studies using libraries derived from hepatocyte-specific eQTLs would improve the functional validation of regulatory variants in liver tissues.

## CONCLUSIONS

Our study highlights the importance of integrating functional genomic data, ancestry-specific analyses, and colocalization approaches to refine the identification of causal regulatory variants in hepatocytes. By leveraging primary hepatocyte eQTLs from an AA cohort, we uncover ancestry-specific regulatory variation that is often overlooked in European-biased datasets. Furthermore, the integration of MPRA data strengthens the functional interpretation of eQTLs, despite differences in cellular context. Future work expanding diverse population cohorts and incorporating additional functional assays within relevant tissues will be crucial for further disentangling the complex genetic architecture of liver gene regulation.

## METHODS

### Primary Hepatocyte Cohort

Seventy-five AA primary hepatocyte lines were acquired, with 59 procured from commercial companies (BioIVT, TRL/Lonza, Life technologies, Corning and Xenotech) and 16 isolated in-house from cadaveric livers, which were obtained through a collaboration with Gift of Hope, who supply non-transplantable organs to scientific researchers. Livers with active cancer or a history of hepatocarcinoma were excluded from the study.

Hepatocytes were isolated from cadaveric livers using a modified two-step collagenase perfusion procedure previously described in Park et. al^60^. Briefly, a lobe was cut from the cadaveric liver and transferred to a perfusion vessel Büchner funnel (Carl Roth) where curved irrigation cannulae with olive tips (Kent Scientific) were then inserted into the larger blood vessels on the exposed cut surface of the liver. The liver section was washed by a three-step perfusion (HEPES buffer-EGTA buffer-HEPES buffer, Sigma-Aldrich) followed by digestion by perfusion (collagenase buffer, Gibco). Once complete, the section was placed in a crystallizing dish (Omnilab) containing 10mM HEPES, 120mM sodium chloride, 6.2 mM potassium chloride, 0.18 mM calcium chloride, and 7.5% bovine serum albumin in water. The Glisson’s capsule was removed, and the tissue was gently shaken to release hepatocytes. The resulting cell suspension was then filtered through a 70 mm nylon mesh, centrifuged at 100 × g for 10 minutes in 4°C, washed and resuspended in plating medium (BioIVT) before assessing cell viability by trypan blue (Lonza) exclusion using a hemocytometer (Fisher Scientific).

During data analysis, certain samples may have been removed during quality control steps for genotyping or RNA-sequencing (Figure 1A).

### Genotyping, Imputation, and Quality Control

#### Genotyping:

DNA was extracted from approximately 1 million cells from each primary hepatocyte line using the Gentra Puregene (Qiagen) kit following manufacturer’s protocol. All DNA samples were bar coded for genotyping. SNP genotyping was conducted using either the Illumina iSelect^®^ 8×1 LCG Custom DNA Analysis Kit (54 samples) or the Illumina Infinium Multi-Ethnic Global Kit (18 samples) using standard protocols. These multi-ethnic global arrays provide superior assaying in non-European populations and improve imputation quality, potentially increasing the number of SNPs used in downstream analysis^61^.

#### Pre-Imputation Quality Control:

The following groups of SNPs were removed from analysis: (1) SNPs located on the sex and mitochondrial chromosomes which would alter minor allele frequency (MAF) values (2) SNPS with a low call rate (<0.95), indicating low genotype quality (3) A/T or C/G SNPs which may introduce flip-strand issues (4) SNPs with a MAF < 0.1. Using PLINK (version 1.9), individuals with discordant sex information were identified using a sex check and duplicated or related individuals were identified using the identity-by-descent (IBD) method^62^. An IBD cutoff score of 0.125 was used, indicating third-degree relatedness or closer. Patient ancestries were confirmed using a principal component analysis (PCA) plot of LD pruned genotype data. The 1000 Genome project was used as reference to identify samples that did not cluster along the spectrum for AA within PCA space^63^.

#### Imputation:

Genotypes were imputed by the TOPMed imputation server (version 1.6.6) using the TOPMed r2 reference panel, GRCh38/hg38 array build, and 0.3 estimated r2 (rsq) filter threshold^64^. Post-imputation QC includes removal of SNPs with poor imputation quality scores (<0.8), failed Hardy-Weinberg equilibrium tests (p < 0.00001), and low MAFs (<0.05). An overview of the genotyping pipeline is shown in Figure 1A.

### RNA-sequencing, Quantification, and Quality Control

#### RNA-sequencing:

Total RNA was extracted from each primary hepatocyte matrigel culture three days after plating using the Qiagen RNeasy Plus mini kit as previously described^65^. Samples with an RNA integrity number (RIN) less than 8 were removed from analysis. Low RIN numbers may be indicative of degraded RNA. Libraries were prepared for sequencing using the TruSeq RNA Sample Prep Kit, Set A (Illumina) per manufacturer’s protocol. The cDNA libraries were prepared and sequenced using either HiSeq2500 (Illumina) or HiSeq4000 (Illumina) instruments by the University of Chicago’s Functional Genomics core, producing single end 50bp reads with approximately 50 million reads per sample. As two instruments were used in this study, we were cognizant of potential batch effect and incorporated methods for correction in the analysis described below.

#### Reads QC:

FastQC (v0.11.2) was used to assess the quality of raw reads from FASTQ files. A per base sequence quality threshold of >20 across all bases was used. Sequence reads were aligned to the human genome sequence GRCh38 and comprehensive gene annotation (GENCODE v.25) using STAR2.5^66,67^. Duplicate mapped reads were removed, and the uniquely mapped reads were indexed by SAMTools(v1.2)^68^. Nucleotide composition bias, GC content distribution, and coverage skewness of the mapped reads were assessed using RNA-SeQC(v.2.6.4)^69^. Samples with nucleotide composition bias, non-normal distribution of GC content, and skewed coverage were removed. Lastly, transcript base distribution was evaluated using Picard CollectRNASeqMetrics within specific genomic regions^70^. Samples with less than 80% of bases aligned to exons and UTR regions were removed. These steps are taken to ensure only reliably mapped reads are included in the downstream analyses. An overview of these quality control steps is shown in Figure 1A.

#### Gene Expression Quantification:

Gene expression was quantified using a collapsed gene model following GTEx isoform collapsing procedure^71^. To evaluate gene-level expression, reads were mapped to genes referenced with GENCODE (v.25) using RNA-SeQC. HTSeq supplied raw counts for gene expression analysis using Bioconductor package DESeq2(v1.20.0)^72,73^. Counts were normalized by regularized log transformation, batch correction was performed using ComBat-Seq, and PCA was performed using DESeq2^74^. PC1 and PC2 were plotted to visualize sample expression patterns; samples whose expression patterns were not clustering with most samples were excluded as outliers. Prior to batch correction, three samples did not cluster with the overall group (Figure S1A). After batch correction, two of these samples clustered with the overall group and were included in analysis, while the third was removed as an outlier (Figure S1B).

Gene expression was normalized by trimmed means of M-values normalization method (TMM) implemented in edgeR^75^. Transcripts per million (TPM) was calculated by first normalizing counts by gene length then by read depth^75,76^.

#### Gene QC:

Gene expression values were filtered based on expression thresholds < 0.1 TPM in at least 20% of samples and ≤ 6 reads in at least 20% of samples.

#### Sample QC:

The expression values for each gene were normalized across samples with inverse normal transformation. An overview of these quality control steps is shown in Figure 1A.

### Expression Quantitative Trait Loci Mapping

*Cis*-eQTL mapping was conducted using data from the 73 AA-hepatocyte samples, which passed QC using tensorQTL with a *cis*-window of 1MB and age, sex, and genomic PC1 as covariates^76^. In addition to these covariates, probabilistic estimation of expression residuals (PEER) factors were calculated from the gene expression data to account for unmeasured confounding variables in transcriptome data^77^. Ten PEER factors were included as covariates.

Significant eQTLs were calculated using the stepwise regression method previously described by GTEx, where nominal p-values are calculated for all *cis*-eQTL candidates followed by adaptive permutation to obtain gene-level p- and q-values^71^. Throughout, eQTLs refer to significant SNP-gene pairs, eSNPs to significant regulatory variants, and eGenes to genes significantly regulated by genetic variation. eGenes were identified at an FDR threshold of 0.10 using q-value<0.10. Significant *cis*-eQTLs for those eGenes were then identified as any variant/gene pair whose nominal p-value was less than the gene-level p-value obtained during permutations.

### Characterization of significant hepatocyte eQTLs

#### Distance to transcriptional start site (TSS):

For each gene included in eQTL analysis, the associated MANE-select transcript was used to define each TSS^78^. The distance between eQTLs and their associated eGene’s TSS is calculated during tensorQTL analysis.

#### Enrichment of eQTLs within ChromHMM active chromatin states:

eQTLs were tested for enrichment within active regulatory regions as defined by ROADMAP’s ChromHMM expanded 18-state chromatin model^19^. The chromatin states considered as active markers were: Active TSS (TssA), Flanking TSS (TssFlnk), Flanking TSS Upstream (TssFlnkU), Flanking TSS Downstream (TssFlnkD), Strong Transcription (Tx), Genic Enhancer 1 (EnhG1), Genic Enhancer 2 (EnhG2), Active Enhancer 1 (EnhA1), Active Enhancer 2 (EnhA2). The genomic locations of these active markers were downloaded for Roadmap liver tissue (E066) and the HepG2 cell line (E118), as these represent the most closely related available datasets to primary human hepatocytes^20^.

To perform the enrichment analysis, we generated 1,000 null sets of SNPs matched to the AAhepatocyte eSNPs based on minor allele frequency (MAF), linkage disequilibrium (LD) score, and distance to the nearest gene’s TSS, stratified into 10 quantile bins. The LD score of each eSNP was calculated using LD Score Regression (LDSC) with a window size of 1cM and the AA-hepatocyte genotype data^79^. We calculated the average number of null SNPs overlapping each ChromHMM active region across 1,000 random sets and assessed enrichment using Fisher’s exact test. The resulting p-values were corrected for multiple testing using the Bonferroni method, and enrichment was reported for annotations with an adjusted p-value < 0.05.

### Comparison to public liver eQTL datasets

Significant eQTLs and all SNP-gene pairs tested in the GTEx liver cohort (version 8) were downloaded from the GTEx portal^80^. The GTEx v8 liver dataset consists of 208 liver samples and includes donors across multiple ancestries. Full marginal eQTL summary statistics from Broadaway *et al*. were downloaded, and significant eQTLs were defined by applying a genome-wide nominal p-value threshold of 1 × 10^−5^. This threshold was selected based on the original study, which demonstrated that it yields a set of significant associations comparable to those identified in GTEx liver, thereby facilitating cross-dataset comparisons^81^. The Broadaway eQTL dataset is a meta-analysis of 1,183 European ancestry samples.

#### Colocalization Analysis:

All comparisons between the AA-hepatocyte eQTL dataset and public liver eQTL datasets were conducted separately for GTEx and Broadaway. For each comparison (AA vs GTEx and AA vs Broadaway), we identified: I. variants tested in both datasets (shared tested variants), II. eQTLs significant in both datasets (overlapping eQTLs), and III. eQTLs significant only in the AA-hepatocyte dataset (AA-specific eQTLs).

Colocalization analysis was performed to evaluate whether eQTL signals for a given eGene in the AA-hepatocyte dataset and each reference dataset were consistent with a shared underlying causal variant.

Analysis was conducted using the R package coloc (v5.2.3) on shared tested variants from each comparison^82^. The variance of the beta coefficient (varbeta) was calculated as the squared standard error of the slope, as recommended for coloc input.

For each comparison, we identified eQTL signals with strong evidence of colocalization (posterior probability of a shared causal variant, PP4 > 0.80), and constructed 95% credible sets of candidate causal variants. Variants were ranked by their posterior probability of being causal (SNP.PP.H4), and credible sets were defined as the minimal set of variants whose cumulative posterior probability exceeded 0.95. Credible sets were defined per eGene and compiled into a combined dataset. Within this study, colocalized variants refer to candidate causal variants identified within the 95% credible sets of colocalized eQTL signals. Overlapping eQTLs without evidence supporting a shared causal variant were labeled as not colocalized. The resulting categories (AA-specific, overlap, colocalized, and not colocalized) were assigned separately for each dataset comparison and used in downstream analyses.

#### Fixation index (Fst) Comparison:

For each significant eQTL, corresponding allele frequencies within African (AFR) and European (EUR) populations were obtained from the 1000 Genomes Project Phase 3^63^. Fst values were calculated using the YRI (Yoruba in Ibadan, Nigeria) and CEU (Utah residents with Northern and Western European ancestry) populations using the GCTA software^83^. Groupings of eQTLs into colocalized, not colocalized, and AA-specific categories were defined based on the comparison between the AA-hepatocyte eQTL dataset and the GTEx liver eQTL dataset. Differences in Fst values between colocalized vs. not colocalized and AA-specific vs. colocalized groups were assessed using the Wilcoxon rank-sum test.

### Comparison to HepG2 Massively Parallel Reporter Assay (MPRA) Active Regulatory Sequences

We compared our AA-hepatocyte eQTL results to an MPRA conducted in the hepatocarcinoma cell line HepG2 to identify regulatory region overlap in a relevant cell type. HepG2 MPRA data was downloaded from the GEO Ascension Browser (GEO: GSE75661) and processed according to Tewhey *et al*., with active sequences defined as any variant which had an effect on the reporter in at least one of the two alleles using an adjusted p-value < 0.01^18^.

We examined variants that were tested in both the AA-hepatocyte eQTL and HepG2 MPRA datasets, identifying those that were significant in both as well as those that were uniquely significant in one dataset. Because MPRAs are inherently not gene-specific, each MPRA-tested variant was matched to all genes it was tested with in the eQTL analysis when assessing overlap between MPRA and eQTL results. Additionally, we assessed the overlap between MPRA-validated variants and those identified through or colocalization analysis of AA-hepatocyte eQTL results and GTEx liver results, providing further insight into putative regulatory variants with functional relevance.

## Supplementary Material

Supplementary Files

This is a list of supplementary files associated with this preprint. Click to download.

- ResultsSuppFile.xlsx
- 22eQTLpaperSUPPFIGURES.docx

## Data Availability

Summary statistics from the *cis*-eQTL and colocalization analysis will be made available on FigShare with publication. Genotyping and expression files will be available from the GEO with publication.
